# The effects of vivifrail-based multicomponent training on physical and cognitive function in frail older adults: a systematic review and meta-analysis

**DOI:** 10.3389/fphys.2025.1646833

**Published:** 2025-07-29

**Authors:** Jiping Chen, Haojie Liu, Haojie Zhao, Jiawei Yao, Yanyu Lu

**Affiliations:** ^1^ School of Physical Education, Shandong University, Jinan, China; ^2^ Culture and Tourism College, Guangdong Vocational Academy of Art, Foshan, Guangdong, China; ^3^ School of Innovation Design, Guangzhou Academy of Fine Arts, Guangzhou, Guangdong, China

**Keywords:** vivifrail, multicomponent training, physical function, cognitive function, frailty

## Abstract

**Objectives:**

Vivifrail is a personalized, multicomponent exercise program that has emerged in recent years. This systematic review and meta-analysis aimed to assess the effects of Vivifrail-based multicomponent training on physical and cognitive function in frail older adults.

**Methods:**

A systematic search was conducted following the PRISMA guidelines, using the following databases: PubMed, Web of Science, The Cochrane Library, EBSCOhost, and Embase, covering studies up to 26 May 2025. The included studies were assessed for quality using the Effective Public Health Practice Project quality assessment tool. Data were analyzed using random-effects meta-analysis.

**Results:**

Twelve studies, including 1,026 participants, were included in the meta-analysis. Vivifrail-based multicomponent training showed significant beneficial effects on the Short Physical Performance Battery (SPPB) (SMD = 0.90; 95% CI: 0.50, 1.30; P < 0.001; I^2^ = 88.1%), grip strength (SMD = 0.62; 95% CI: 0.37, 0.87; P < 0.001; I^2^ = 68.9%), and cognitive function (SMD = 0.60; 95% CI: 0.34, 0.86; P < 0.001; I^2^ = 56.9%) compared to the usual care group. No significant effects were observed on the Barthel Index of activities of daily living (ADL) (SMD = 0.87; 95% CI: −0.12, 1.85; P = 0.083; I^2^ = 95.7%). Furthermore, no significant risk of publication bias was detected for any outcomes.

**Conclusion:**

Current evidence suggests that Vivifrail-based multicomponent training significantly improves physical and cognitive function. However, the results should be interpreted with caution due to high heterogeneity in the meta-analysis.

**Systematic Review Registration:**

identifier CRD420251071483.

## 1 Introduction

Frailty is an age-associated condition characterized by diminished physiological reserves associated with chronic illnesses, falls, fractures, limited mobility in older persons, and eventually, mortality (Dent et al., 2019; Fried et al., 2001; Clegg et al., 2013; Xue, 2011; Fan et al., 2020; Wang et al., 2023; Morley, 2013). To date, millions of older adults worldwide suffer from frailty, and this number is still growing due to the ageing population (Hoogendijk et al., 2019). This prevalence of frailty among older adults is undoubtedly placing increasing pressure on global healthcare systems. Notably, the processes that contribute to frailty in older adults may also play a role in limited mobility and cognitive impairment (Ward et al., 2021). It significantly affects individuals, their families, and society, with an annual direct healthcare cost of $17,700 per person (Wimo et al., 2007). Accordingly, effectively managing functional deterioration and cognitive decline in the frail elderly population has become a critical issue that requires urgent attention.

Aerobic exercise, resistance training, and stability exercises individually contribute to improvements in muscle strength, cardiorespiratory endurance, and balance among frail older adults (García et al., 2019; Ferraro et al., 2019). However, multi-component exercise, which integrates various forms of physical activity, demonstrates even greater potential and has garnered significant attention in both recent research and clinical practice (Song et al., 2023; Linhares et al., 2022). Among them, Vivifrail is a multicomponent exercise program tailored to the individual, designed to ease the prescription of evidence-based exercise interventions in older people (Izquierdo et al., 2016). In 2024, it was included in the Integrated Care of Elderly People (ICOPE) guidelines developed by the World Health Organization (WHO) as a treatment option for individuals with limited mobility (Organisation WHO, 2024). Several research studies have demonstrated that the Vivifrail project provides a range of benefits for older adults experiencing frailty, including improved body composition, cardiorespiratory fitness, and quality of life (Romero-García et al., 2021; Hartantri et al., 2023; Martínez-Velilla et al., 2021). Such improvements can be attributed to the cumulative advantages of various forms of physical activity, which promote better mitochondrial function, increased expression of brain-derived neurotrophic factor (BDNF), and neuroprotective effects stemming from vascular neurogenesis (Burtscher et al., 2021). Despite these benefits, little is known about the effect of the Vivifrail project on physical and cognitive functioning. A randomized controlled trial (RCT) revealed that participants in the Vivifrail project exhibited better physical and cognitive performance compared to the usual care group (Casas-Herrero et al., 2022). However, another small-scale study conducted by chen et al. shows 12 weeks of the Vivifrail project did not improve cognitive function significantly (Chen et al., 2023).

To address these research gaps, we conducted a comprehensive systematic review and meta-analysis that included related published studies, with no limit on study type or language restriction. The objective is to investigate the impact of Vivifrail-based multicomponent training on physical and cognitive function in frail older adults.

## 2 Methods

### 2.1 Study registration and protocol

This systematic review and meta-analysis followed the Preferred Reporting Items for Systematic Review and Meta-Analysis (PRISMA) guidelines (Moher et al., 2009). The PRISMA Checklist is available in the Supplementary Table S1. The present research has been registered at the International Prospective Register of Systematic Reviews (PROSPERO) with the protocol number CRD420251071483.

### 2.2 Information sources and search strategy

A literature search was conducted on 12 May 2024, with an updated search on 26 May 2025, using the following databases: PubMed, Web of Science, The Cochrane Library, EBSCOhost, and Embase. The search strategy for each database were identified based on previous original research and combined the following relevant terms: old adults OR frailty AND Vivifrail AND motor ability OR cognitive function. The whole search strategy is described in the Supplementary Table S2.

### 2.3 Eligibility criteria

The eligibility criteria for this review were structured according to the PICOS (Patient/Population, Intervention, Comparison, Outcome, and Study design) framework (Spitzer et al., 1978): (1) participants: frailty adults aged 65 years or older, regardless of sex; (2) intervention: the intervention content is based on the recently developed Vivifrail multicomponent exercise programme (https://vivifrail.com/; Izquierdo, 2019). The Vivifrail is a home exercise program designed for older adults. It focuses on creating personalized exercise plans based on each individual’s abilities. The program includes different types of exercises, such as strength training, balance, flexibility, and cardiovascular activities like walking; (3) comparison: the control group received usual care or maintained their habits without any intervention; (4) outcomes: an assessment of at least one of the following motor ability or cognitive function in humans: the short physical performance battery (SPPB), the Barthel Index of activities of daily living (ADL), the grip strength of the dominant hand, the score of Mini-Mental State Examination (MMSE) and Montreal Cognitive Assessment (MOCA); (5) study design: in order to maximise the inclusion of high quality, we included randomized controlled trials (RCT), clinical trials (CT) and quasi-experiment study.

A total of two reviewers (J.C. and J.Y.) were involved in the independent assessment of the title, abstract and full text for eligibility for inclusion. If necessary, a third investigator (H.Z.) was consulted. When assessing the full texts, we used Kappa consistency to assess whether the two reviewers’ judgements of the literature were consistent.

### 2.4 Data collection process

Data for each study were retrieved independently by two assessors (J.C. and J.Y.), and the following information was collected: (1) surname of the primary author; (2) year of publication; (3) country of the study; (4) type of study design; (5) number, sex and age of participants in each of the intervention and control groups; (6) characteristics of interventions concerning content, frequency, duration of each exercise mode; (7) the related outcomes of motor ability or cognitive function; (8) the reported mean pre-intervention (Mpre), the reported mean post-intervention (Mpost), the standard deviation from the pre-intervention (SDpre), the standard deviation from the post-intervention (SDpost), and sample size (N) recorded for each group prior to and following the intervention. The data were used to calculate the differences of mean (Mdiff) and standard deviation (SDdiff) in each outcome indicator before and after the intervention. The calculations were executed as detailed below:

$$M_{\text{diff}}{=M}_{\text{post}}-M_{\text{Pre}}$$

$$\text{SDdiff}=\sqrt{{SDpre}^{2}+{SDpost}^{2}-2r+SDpre\times SDpost}$$

If the study exclusively reported standard error (SE) or confidence intervals (CIs), they were transformed into standard deviation (SD) utilizing the following formula:

$$SD=SE\times \sqrt{N}$$

$$SD=\sqrt{N}\times \frac{CIupper-CIlower}{2t}$$

In this context, CIupper refers to the higher limit of the confidence interval, CIlower signifies the lower limit of the confidence interval, and t corresponds to the t distribution with N – 1 degrees of freedom for the appropriate confidence interval (Cumpston et al., 2019). The details of outcome data for each study are available in the Supplementary Table S3.

### 2.5 Risk of bias of individual studies

Two authors, J.C. and J.Y., independently evaluated the risk of bias in the included studies using the Cochrane Risk of Bias Instrument (Higgins et al., 2011). Studies were assessed for quality on six criteria: (1) random sequence generation; (2) allocation concealment; (3) blinding of participants and personnel; (4) blinding of outcome assessment; (5) incomplete outcome data; (6) selective reporting and (7) other bias. In addition, two independent researchers (J.C. and Y.L.) used the Grading of Recommendations Assessment, Development, and Evaluation (GRADE) to evaluate the level of evidence of the meta-analysis (Guyatt et al., 2011).

### 2.6 Data synthesis and analysis

All analyses were performed in Stata software (v14.0; StataCorp, College Station, TX, United States). Given the expected heterogeneity among the studies, the meta-analysis was performed using a generic inverse variance pooling method and pooled effect sizes with a random-effects model using the random-effects model (DerSimonian and Laird approach) to summarize the effects of VI on motor ability or cognitive function to compare to control group (Jackson et al., 2010). As all outcomes of interest were continuous and potentially susceptible to slight sample bias, the standard mean difference (SMD) with estimated Hedges’ g was selected as the effect estimate instead of Cohen’s d (Cumpston et al., 2019). The polled effect sizes for Hedges’ g were classified as small (0 ≤ g ≤ 0.50), moderate (0.50 < g ≤ 0.80) and large (>0.80) (Cohen, 2013). Statistical significance was set at p < 0.05.

Heterogeneity among studies was evaluated using the inconsistency index (I^2^), which is based on the Cochran Q statistic (Higgins and Thompson, 2002). The I^2^ statistic is the proportion of the observed variance due to the actual between-study variance. Values of 25%, 50% and 75% might be considered as low, moderate and high, respectively. In search of a source of heterogeneity, we selected the following variables for subgroup analyses: (1) study design, (2) age, and (3) duration of training. Publication bias in our study was assessed with Egger regression test and funnel plots, the p > 0.05 was considered without any publication bias (Egger et al., 1997). To further ensure the robustness of the bias results, we used Duval and Tweedie’s trim and fill method for further verification (Duval and Tweedie, 2000).

In addition, random-effects meta-regression analyses were conducted to assess whether the results might vary according to differences in study design, age, and duration of training between the intervention and control groups.

## 3 Results

### 3.1 Study selection

**FIGURE 1:**
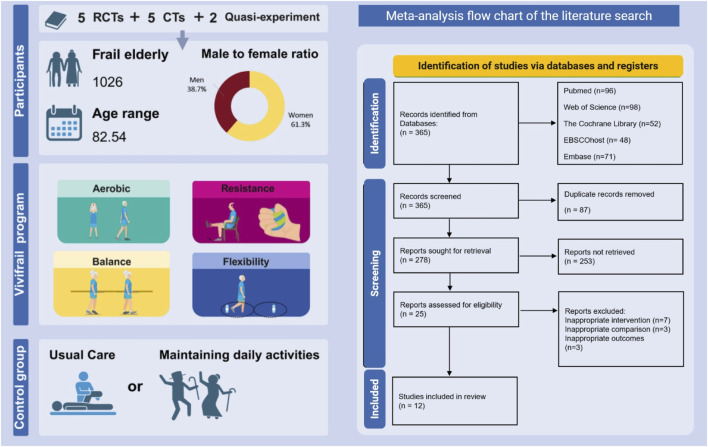
The study overview and PRISMA flow diagram in the meta-analysis.

The electronic search strategy retrieved 365 records (PubMed = 96; Web of Science = 98; The Cochrane Library = 52; EBSCOhost = 48; Embase = 71). After removing duplicate references and screening titles and abstracts, 278 articles were excluded. Of the remaining 278 articles, after full-text screening and checking the reference lists of included studies, 25 were read in full. The reasons for exclusion based on the full text were (1) inappropriate intervention (7 articles), (3) Inappropriate comparison (3 articles), and (4) Inappropriate outcomes (3 articles). The Kappa results indicate that the two reviewers’ opinions are highly consistent (P = 0.84, Supplementary Table S4). Therefore, 12 studies were included in the final systematic review and meta-analysis (Romero-García et al., 2021; Martínez-Velilla et al., 2021; Casas-Herrero et al., 2022; Chen et al., 2023; Sánchez-Sánchez et al., 2022; Gutiérrez-Reguero et al., 2024; Dobarro et al., 2023; Li et al., 2025; Sáez de Asteasu et al., 2024; Mawadda et al., 2024; Soenarti et al., 2024; Courel-Ibáñez et al., 2021). The study overview and PRISMA flow diagram in the meta-analysis is shown in Figure 1.

### 3.2 Study characteristics

Table 1 presents the characteristics of the included studies. Among the 12 studies, 5 articles were RCT (Martínez-Velilla et al., 2021; Casas-Herrero et al., 2022; Gutiérrez-Reguero et al., 2024; Dobarro et al., 2023; Li et al., 2025), 5 articles were clinical trial (CT) (Romero-García et al., 2021; Chen et al., 2023; Sánchez-Sánchez et al., 2022; Sáez de Asteasu et al., 2024; Courel-Ibáñez et al., 2021), and 2 articles were quasi-experiment (Mawadda et al., 2024; Soenarti et al., 2024). 12 studies were from 5 countries: Spain (6 studies) (Martínez-Velilla et al., 2021; Casas-Herrero et al., 2022; Gutiérrez-Reguero et al., 2024; Dobarro et al., 2023; Sáez de Asteasu et al., 2024; Courel-Ibáñez et al., 2021), China (2 studies) (Chen et al., 2023; Li et al., 2025), Indonesia (2 studies) (Mawadda et al., 2024; Soenarti et al., 2024), France (1 study) (Sánchez-Sánchez et al., 2022), and Mexico (1 study) (Romero-García et al., 2021). Publication dates ranged from 2020 to 2025. The final analysis comprised 1,026 adults (61.3% female) with an average age of 82.54.

**TABLE 1:** Characteristics of included studies.

Study	Country	Design	Sample size	Age range (mean ± SD)	Women, N (%)	Outcomes
Casas-Herrero et al. (2022)	Spain	RCT	EX: 88 UC: 100	EX: 84.2 ± 4.8 UC: 84.0 ± 4.8	EX: 63 (71.6) UC: 69 (69.0)	(1): SPPB; (2): MOCA; (3): ADL; (4): Hand grip (kg).
Chen et al. (2023)	China	CT	EX: 44 UC: 60	EX: 83 ± 1.5 UC: 86 ± 1.75	EX: 26 (59) UC: 33 (55)	(1): SPPB; (2): MMSE; (3): ADL; (4): Handgrip (kg).
Courel-Ibáñez et al. (2021)	Spain	Clinical trial	EX: 12 UC: 12	EX: 87.2 ± 6.5 UC: 87.3 ± 7.7	14 (58.3)	(1): SPPB; (2): Handgrip (kg).
Dobarro et al. (2023)	Spain	RCT	EX: 29 UC: 27	EX: 83.2 ± 5.1 UC: 82.6 ± 5.7	12 (21.4)	(1): SPPB.
Gutiérrez-Reguero et al. (2024)	Spain	RCT	EX: 17 UC: 19	EX: 86.2 ± 8.9 UC: 78.2 ± 9.8	EX: 12 (70.6) UC: 14 (73.7)	(1): SPPB; (2): MMSE; (3): ADL; (4): Handgrip (kg).
Li et al. (2025)	China	Randomized controlled trial	EX: 30 UC: 29	EX: 75.33 ± 3.07 UC: 75.17 ± 3.59	EX: 19 (63.3) UC: 16 (55.2)	(1): SPPB; (2): ADL; (3): Handgrip (kg).
Martínez-Velilla et al. (2021)	Spain	Randomized controlled trial	EX: 54 UC: 49	EX: 87 ± 4 UC: 86 ± 5	EX: 25 (46.3) UC: 28 (57.1)	(1): SPPB; (2): MMSE; (3): ADL; (4): Handgrip (kg).
Mawadda et al. (2024)	Indonesia	Quasi-experiment	EX: 25 UC: 25	EX: 72.56 ± 9.65 UC: 71.96 ± 8.34	EX: 13 (52) UC: 14 (56)	(1): SPPB; (2): Handgrip (kg).
Romero-García et al. (2021)	Mexico	CT	EX: 33 UC: 28	EX: 71.43 ± 5.95 UC: 70.15 ± 3.65	61 (100)	(1): SPPB; (2): Handgrip (kg).
Sáez de Asteasu et al. (2024)	Spain	Clinical trial	EX: 64 UC: 66	EX: 87.1 ± 4.7 UC: 88.4 ± 4.5	EX: 27 (42.2) UC: 30 (45.5)	(1): SPPB; (2): Handgrip (kg).
Sánchez-Sánchez et al. (2022)	France	CT	EX: 88 UC: 100	EX: 84.2 ± 4.8 UC: 84.0 ± 4.8	EX: 60 (76.92) UC: 60 (68.18)	(1): SPPB; (2): MOCA; (3): Handgrip (kg).
Soenarti et al. (2024)	Indonesia	Quasi-experiment	EX: 17 UC: 10	EX: 67.47 ± 6.61 UC: 72.20 ± 3.91	EX: 11 (64.7) UC: 8 (80)	(1): MOCA.

All intervention components in 12 studies were based on the Vivifrail multicomponent intervention and consisted of four components: aerobic, progressive resistance, balance, and flexibility training. All of these studies used the native language of the country where the study was conducted to communicate the content of the intervention to participants. The content focuses on exercises for lower-limb muscles, including seated squats, leg presses, and bilateral knee extensions, as well as upper body workouts like the seated bench press. It also includes balance and gait retraining activities, such as semi-tandem walking, single-leg standing, stepping practice, and navigating small obstacles. Proprioceptive exercises on unstable surfaces, along with weight transfer drills between legs, are also included. Most studies had a 12-week intervention period, with three to five interventions administered per week. Details about the content of the intervention program are presented in Supplementary Table S5.

The outcome indicators in this study included physical function measures (SPPB, ADL, and grip strength) as well as cognitive function assessments (MMSE, MOCA). Most of the studies incorporated at least two outcome metrics. Specifically, 11 studies provided data for SPPB (Romero-García et al., 2021; Martínez-Velilla et al., 2021; Casas-Herrero et al., 2022; Chen et al., 2023; Sánchez-Sánchez et al., 2022; Gutiérrez-Reguero et al., 2024; Dobarro et al., 2023; Li et al., 2025; Sáez de Asteasu et al., 2024; Mawadda et al., 2024; Courel-Ibáñez et al., 2021), 6 studies focused on cognitive function (Martínez-Velilla et al., 2021; Casas-Herrero et al., 2022; Chen et al., 2023; Sánchez-Sánchez et al., 2022; Gutiérrez-Reguero et al., 2024; Soenarti et al., 2024), 5 studies addressed ADL (Martínez-Velilla et al., 2021; Casas-Herrero et al., 2022; Chen et al., 2023; Gutiérrez-Reguero et al., 2024; Li et al., 2025), and 10 studies examined grip strength (Romero-García et al., 2021; Martínez-Velilla et al., 2021; Casas-Herrero et al., 2022; Chen et al., 2023; Sánchez-Sánchez et al., 2022; Gutiérrez-Reguero et al., 2024; Sáez de Asteasu et al., 2024; Mawadda et al., 2024; Courel-Ibáñez et al., 2021).

### 3.3 Risk of bias within studies

The summary results of the Cochrane risk of bias assessment tool are shown in Supplementary Figure S1. Two studies had a high risk of bias in terms of blinding of participants and researchers (Casas-Herrero et al., 2022; Courel-Ibáñez et al., 2021), two studies had a high risk of bias in terms of incomplete outcome data (Sánchez-Sánchez et al., 2022; Soenarti et al., 2024), and three studies had a high risk of bias in terms of selective reporting (Dobarro et al., 2023; Li et al., 2025; Soenarti et al., 2024). Additionally, the results of the GRADE assessment show that the quality of evidence from the meta-analysis is acceptable (Supplementary Figure S2).

### 3.4 Main results

**FIGURE 2:**
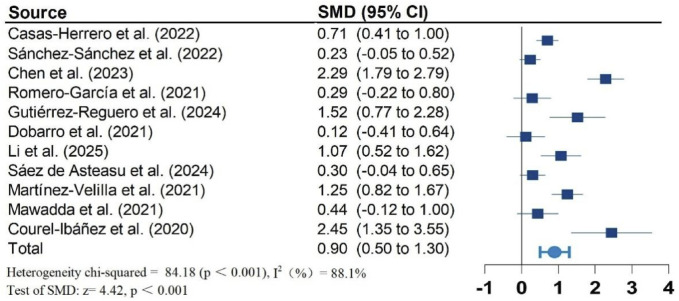
Effect of Vivifrail multicomponent training on SPPB.

**FIGURE 3:**
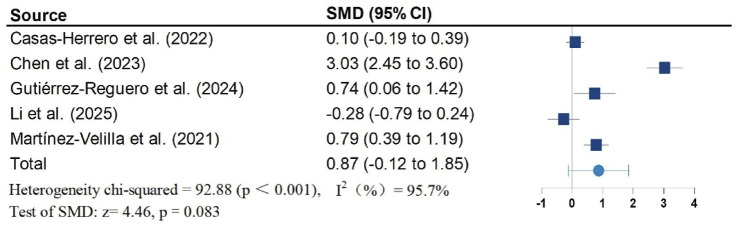
Effect of Vivifrail multicomponent training on ADL.

**FIGURE 4:**
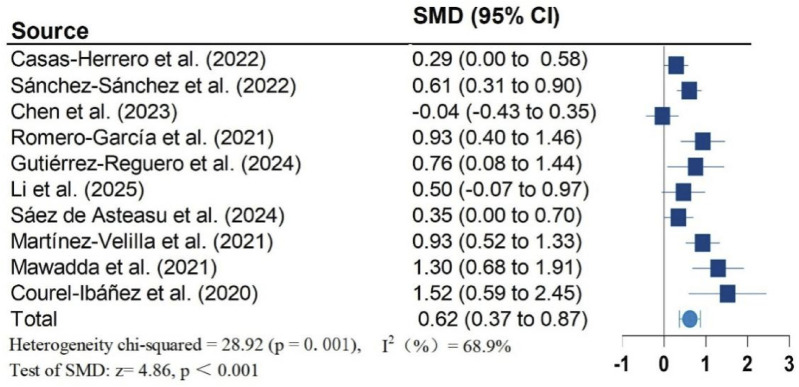
Effect of Vivifrail multicomponent training on hand grip.

**FIGURE 5:**
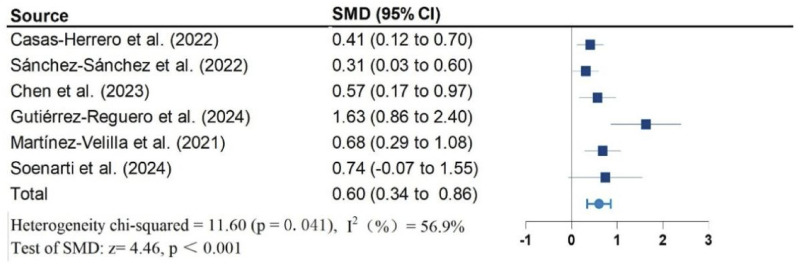
Effect of Vivifrail multicomponent training on cognitive function.

Figures 2–5 present the forest plot of the effects of VI on four outcomes. The results of meta-analysis show that compared with the control conditions, VI interventions were associated with significant increases in SPPB (SMD = 0.90; 95% CI: 0.50, 1.30; P < 0.001; I^2^ = 88.1%), cognitive function (SMD = 0.60; 95% CI: 0.34, 0.86; P < 0.001; I^2^ = 56.9%), and handgrip strength (SMD = 0.62; 95% CI: 0.37, 0.87; P < 0.001; I^2^ = 68.9%). However, there is no statistically significant differences of VI on ADL (SMD = 0.87; 95% CI: −0.12, 1.85; P = 0.083; I^2^ = 95.7%).

Due to significant heterogeneity in all results. To identify the source of heterogeneity, subgroup analyses were performed. The results of the subgroup analyses showed no heterogeneity in the effects of VI on SPPB and grip strength in frail older adults aged greater than or equal to 80 years. There was also no heterogeneity in the results of VI improving cognitive function in frail older adults in non-RCT studies. The results of subgroup analysis in the meta-analysis are shown in Table 2.

**TABLE 2:** The results of subgroup analysis.

Outcome			N	SMD (95% CI)	I^2^ (%)	P value (heterogeneity)
Subgroup						
SPPB	Design	RCT	442	0.90 (0.48–1.32)	74.10%	<0.001
		non-RCT	560	0.93 (0.24–1.61)	92.30%	0.004
	Age	<80	197	0.60 (0.13–1.06)	91.10%	<0.001
		≥80	905	1.03 (0.51–1.55)	56.00%	0.1
	Duration	<12 weeks	130	0.304 (−0.04–0.65)	NA	NA
		≥12 weeks	872	0.98 (0.53–1.42)	88.50%	<0.001
Cognition	Design	RCT	327	0.79 (0.26–1.33)	77.20%	0.012
		non-RCT	319	0.43 (0.20–0.65)	0.00%	0.433
	Age	<80	27	0.73 (−0.07–1.55)	NA	NA
		≥80	619	0.59 (0.31–0.88)	64.60%	0.023
	Duration	<12 weeks	—	—	—	—
		≥12 weeks	—	—	—	—
ADL	Design	RCT	286	0.32 (−0.16–0.80)	78.50%	0.003
		non-RCT	104	3.03 (2.45–3.60)	NA	NA
	Age	<80	—	—	—	—
		≥80	—	—	—	—
	Duration	<12 weeks	—	—	—	—
		≥12 weeks	—	—	—	—
Hand grip	Design	RCT	386	0.57 (0.24–0.91)	55.40%	0.081
		non-RCT	557	0.68 (0.29–1.07)	77.40%	<0.001
	Age	<80	170	0.53 (0.25–0.81)	69.80%	0.003
		≥80	773	0.87 (0.40–1.34)	54.80%	0.11
	Duration	<12 weeks	154	0.61 (0.33–0.89)	70.20%	0.001
		≥12 weeks	789	0.85 (−0.29–1.99)	81.40%	0.02

Furthermore, meta-regression analyses were conducted to explore the modifying effects of study design, age, and total training duration. But we did not find a significant association between any other variables of exercise and the effects of VI on SPPB, cognitive function, ADL, and handgrip strength (p > 0.05 for all). Details of the statistical results are available in Supplementary Table S6.

### 3.5 Publication bias

In the overall results, there is no risk of publication bias was found in the meta-analysis for SPPB (Egger’s P value = 0.72), cognitive function (Egger’s P value = 0.64), ADL (Egger’s P value = 0.60), and hand grip (Egger’s P value = 0.71) (Supplementary Table S7). Also, the funnel plots for all outcomes were approximately symmetrical, suggesting no significant publication bias (Supplementary Figure S3). Supplementary Table S7 shows that the results are consistent or change very little in effect sizes before and after the correction, suggesting that the results are robust and that bias may not have had a significant impact on the overall conclusions about effect sizes.

## 4 Discussion

This systematic review and meta-analysis provided the first comprehensive evaluation of the impact of Vivifrail-based multicomponent training on physical and cognitive function in frail older adults. A total of 12 studies, involving 1,026 participants, were systematically reviewed. Among the four outcomes assessed, significant beneficial effects were identified for SPPB, cognitive function, and hand grip strength. However, no significant impact was observed on ADL.

In recent years, accumulating evidence has shown that multicomponent training is beneficial to physical function in older adults. A meta-analysis comprising 13 studies demonstrated that multicomponent training enhances physical function in healthy older adults by increasing whole-body strength, walking speed, and aerobic capacity (Labata-Lezaun et al., 2023). Another meta-analysis of 14 studies by Linhares et al. showed that 27 weeks of multicomponent training in older women with osteoporosis improved strength and functional health and reduced the risk of falls (Linhares et al., 2022). Moreira et al., Halvarson et al., and Carter et al. further verified similar results in RCTs (Moreira et al., 2021; Carter et al., 2002; Halvarsson et al., 2015). However, current research on Vivifrail is inadequate. An RCT involving 188 frail older adults demonstrated the potential for Vivifrail to raise physical function (Sánchez-Sánchez et al., 2022). The results of this study showed a significant increase in SPBB in the Vivifrail group compared to the control group (β = 0.42; 95% CI = 0.10, 0.74; P < 0.001). In another RCT study, Chen et al. intervened with 104 frail older adults who were randomly assigned to participate in either the 12-week home-based, individualized Vivifrail program or usual care (Chen et al., 2023). After 8 weeks of intervention, assessed using the SPPB, participants showed a slight but significant improvement in physical function. Consistent with the literature above, our meta-analysis revealed that Vivifrail had beneficial effects on SPPB and grip strength compared to the control group. These results further support the existing evidence suggesting that performing Vivifrail helps improve physical function in frail older adults.

The benefits of Vivifrail in frail older adults may be related to the upregulation of local expression of insulin-like growth factor 1 (IGF-1) in skeletal muscle (Chen et al., 2024). As a crucial driver of muscle growth and regeneration, IGF-1 not only induced skeletal myotube hypertrophy through PtdIns-3-OH kinase (PI (Clegg et al., 2013) K)/Akt/mammalian target of rapamycin (mTOR) and PI (Clegg et al., 2013) K/Akt/glycogen synthase kinase 3 (GSK3) pathways (Rommel et al., 2001). It can also inhibit the transcriptional upregulation of key mediators involved in skeletal muscle atrophy, specifically the ubiquitin ligases MuRF1 and MAFbx (Glass, 2005). Additionally, the improvement of physical function is influenced by a range of factors, including age, gender, diet, and lifestyle (Nunes et al., 2022; Zhu et al., 2022). This explains why, in the results of this research, Vivifrail had a limited effect on SPPB and grip strength or even failed to affect ADL. Given that inactivity lifestyles in older adults can lead to an early loss of physical function, Vivifrail should be maintained for a longer period. Therefore, more research on the long-term effects of Vivifrail is needed in the future.

Exercise plays a crucial role in enhancing the cognitive abilities of the elderly population. This enhancement can be attributed to exercise’s ability to increase the levels of neurotrophins, particularly brain-derived neurotrophic factor (BDNF), which supports cell survival, neurite outgrowth, and synaptic plasticity (Coelho et al., 2014; Cotman and Berchtold, 2002; Stigger et al., 2019). Furthermore, regular physical activity improves cerebrovascular and endothelial function, reduces oxidative stress and systemic inflammation, and aids in regulating immune function (Phillips et al., 2015; Walsh et al., 2011). Collectively, these benefits may help prevent or reverse age-related neuronal dysfunction. To evaluate cognitive function, tools such as the MMSE and MoCA are widely used (Folstein et al., 1975; Nasreddine et al., 2005). An increasing number of studies are employing both assessments as cognitive screening tools for older adults. For instance, one systematic review and meta-analysis involving 909 elderly individuals with Alzheimer’s disease found that aerobic exercise increased the overall MMSE score by 1.50 (weighted mean difference), which is clinically significant for Alzheimer’s patients (Zhang et al., 2022). In another study, a meta-analysis focusing on adults aged 60 and older examined the effect of multi-component training on cognitive function in elderly individuals without cognitive impairment (Borges-Machado et al., 2021). This study utilized a simple mental state assessment along with the MoCA to evaluate overall cognitive function. The findings revealed that compared to the control group, multi-component training significantly improved cognitive function (standardized mean difference = 0.58, 95% CI: 0.34–0.81, I^2^ = 11%; P < 0.001). Despite these promising findings, a systematic review or meta-analysis investigating the impact of Vivifrail on cognitive function has not yet been conducted. Our study seeks to fill this gap. We found that Vivifrail interventions administered three to five times a week over approximately 12 weeks can lead to significant improvements in cognitive function. While these results are consistent with previous clinical findings, the cognitive changes observed in this model are modest. Therefore, future research should focus on the clinical significance of Vivifrail in relation to cognitive function in frail elderly individuals.

Although it is the first systematic review and meta-analysis examining the impact of Vivifrail intervention’s impact on physical and cognitive function in frail older adults. However, some limitations should be acknowledged. First, we conducted a comprehensive study of mainstream databases, but were limited by the small number of studies. We included only 12 papers with 1,026 frail older adults. Follow-up studies should use designs with larger sample sizes to improve the precision and external validity of the results. Secondly, despite our attempts to explain heterogeneity using meta-regression and subgroup analysis, the diversity of study designs (randomised controlled trials, controlled trials, quasi-experimental studies) and the complexity and multidimensional nature of frailty, particularly when associated with multiple diseases, remain significant challenges. Our meta-analysis results still exhibit significant heterogeneity. Therefore, results must be interpreted with caution, as these differences may have a significant impact on the outcomes of the Vivifrail intervention. Lastly, the duration of the included Vivifrail intervention ranged from 1 to 24 weeks, with a single trial extending to 6 months. Considering the necessity for optimal efficacy in clinical and public health practices, it is essential that the health benefits of long-term exercise interventions be addressed in future large-scale randomized controlled trials, despite organizations like the National Institute for Health and Care Excellence (NICE) primarily emphasizing RCTs with a minimum follow-up of 12 months.

## 5 Conclusion

This systematic review and meta-analysis investigated the effects of Vivifrail-based multicomponent training on physical and cognitive function in frail older adults. SPPB, ADL, and hand grip strength were used as outcome measures for physical function, while MMSE and MOCA served as proxies for cognitive function. We employed an algorithmic strategy to standardize and integrate the diverse data, allowing for a unified comparison of the impact of Vivifrail. By evaluating 12 trials covering four distinct health outcomes, we found that Vivifrail-based multicomponent training significantly improved physical function (SPPB, hand grip) and cognitive function, but did not affect ADL compared to usual care. These results address a research gap regarding the role of such programs in enhancing physical and cognitive health in frail older adults, and provide a solid theoretical foundation for clinical research and policy development.

## Data Availability

The original contributions presented in the study are included in the article/Supplementary Material, further inquiries can be directed to the corresponding author.

## References

[B1] Borges-Machado F. Silva N. Farinatti P. Poton R. Ribeiro Ó. Carvalho J. (2021). Effectiveness of multicomponent exercise interventions in older adults with dementia: a meta-analysis. Gerontologist 61 (8), e449–e462. 10.1093/geront/gnaa091 32652005 PMC8599205

[B2] Burtscher J. Millet G. P. Place N. Kayser B. Zanou N. (2021). The muscle-brain axis and neurodegenerative diseases: the key role of mitochondria in exercise-induced neuroprotection. Int. J. Mol. Sci. 22 (12), 6479. 10.3390/ijms22126479 34204228 PMC8235687

[B3] Carter N. D. Khan K. M. McKay H. A. Petit M. A. Waterman C. Heinonen A. (2002). Community-based exercise program reduces risk factors for falls in 65- to 75-year-old women with osteoporosis: randomized controlled trial. CMAJ Can. Med. Assoc. J. = J. de l'Association medicale Can. 167 (9), 997–1004. PMC134175 12403738

[B4] Casas-Herrero Á. Sáez de Asteasu M. L. Antón-Rodrigo I. Sánchez-Sánchez J. L. Montero-Odasso M. Marín-Epelde I. (2022). Effects of vivifrail multicomponent intervention on functional capacity: a multicentre, randomized controlled trial. J. cachexia, sarcopenia muscle 13 (2), 884–893. 10.1002/jcsm.12925 35150086 PMC8977963

[B5] ChenB. LiM. ZhaoH. LiaoR. LuJ. TuJ. (2023). Effect of multicomponent intervention on functional decline in Chinese older adults: a multicenter randomized clinical trial. J. Nutr. health and aging 27 (11), 1063–1075. 10.1007/s12603-023-2031-9 37997729

[B6] Chen J. Jia S. Guo C. Fan Z. Yan W. Dong K. (2024). Research progress on the effect and mechanism of exercise intervention on sarcopenia obesity. Clin. interventions aging 19, 1407–1422. 10.2147/CIA.S473083 PMC11319865 39139211

[B7] Clegg A. Young J. Iliffe S. Rikkert M. O. Rockwood K. (2013). Frailty in elderly people. Lancet London, Engl. 381 (9868), 752–762. 10.1016/S0140-6736(12)62167-9 PMC4098658 23395245

[B8] Coelho F. G. Vital T. M. Stein A. M. Arantes F. J. Rueda A. V. Camarini R. (2014). Acute aerobic exercise increases brain-derived neurotrophic factor levels in elderly with alzheimer's disease. J. Alzheimer's Dis. JAD. 39 (2), 401–408. 10.3233/JAD-131073 24164734

[B9] Cohen J. (2013). Statistical power analysis for the behavioral sciences. routledge.

[B10] Cotman C. W. Berchtold N. C. (2002). Exercise: a behavioral intervention to enhance brain health and plasticity. Trends Neurosci. 25 (6), 295–301. 10.1016/s0166-2236(02)02143-4 12086747

[B11] Courel-Ibáñez J. Pallarés J. G. García-Conesa S. Buendía-Romero Á. Martínez-Cava A. Izquierdo M. (2021). Supervised exercise (vivifrail) protects institutionalized older adults against severe functional decline after 14 weeks of COVID confinement. J. Am. Med. Dir. Assoc. 22 (1), 217–9.e2. 10.1016/j.jamda.2020.11.007 33296679 PMC7837301

[B12] Cumpston M. Li T. Page M. J. Chandler J. Welch V. A. Higgins J. P. (2019). Updated guidance for trusted systematic reviews: a new edition of the cochrane handbook for systematic reviews of interventions. Cochrane database Syst. Rev. 10 (10), Ed000142. 10.1002/14651858.ED000142 31643080 PMC10284251

[B13] Dent E. Martin F. C. Bergman H. Woo J. Romero-Ortuno R. Walston J. D. (2019). Management of frailty: opportunities, challenges, and future directions. Lancet London, Engl. 394 (10206), 1376–1386. 10.1016/S0140-6736(19)31785-4 31609229

[B14] Dobarro D. Costas-Vila A. Melendo-Viu M. Cordeiro-Rodríguez M. Íñiguez-Romo A. Rodríguez-Pascual C. (2023). Home exercise intervention with the vivifrail program in frail older patients with heart failure with reduced ejection fraction. The ExFRAIL-HF randomized trial. Rev. espanola Cardiol. English 76 (11), 939–943. 10.1016/j.rec.2023.06.001 37315922

[B15] Duval S. Tweedie R. (2000). Trim and fill: a simple funnel-plot-based method of testing and adjusting for publication bias in meta-analysis. Biometrics 56 (2), 455–463. 10.1111/j.0006-341x.2000.00455.x 10877304

[B16] Egger M. Smith G. D. Schneider M. Minder C. (1997). Bias in meta-analysis detected by a simple, graphical test. bmj 315 (7109), 629–634. 10.1136/bmj.315.7109.629 9310563 PMC2127453

[B17] Fan J. Yu C. Guo Y. Bian Z. Sun Z. Yang L. (2020). Frailty index and all-cause and cause-specific mortality in Chinese adults: a prospective cohort study. Lancet Public health 5 (12), e650–e660. 10.1016/S2468-2667(20)30113-4 33271078 PMC7708389

[B18] Ferraro F. V. Gavin J. P. Wainwright T. McConnell A. (2019). The effects of 8 weeks of inspiratory muscle training on the balance of healthy older adults: a randomized, double-blind, placebo-controlled study. Physiol. Rep. 7 (9), e14076. 10.14814/phy2.14076 31074198 PMC6509064

[B19] Folstein M. F. Folstein S. E. McHugh P. R. (1975). Mini-mental state. A practical method for grading the cognitive state of patients for the clinician. J. psychiatric Res. 12 (3), 189–198. 10.1016/0022-3956(75)90026-6 1202204

[B20] Fried L. P. Tangen C. M. Walston J. Newman A. B. Hirsch C. Gottdiener J. (2001). Frailty in older adults: evidence for a phenotype. journals gerontology Ser. A, Biol. Sci. Med. Sci. 56 (3), M146–M156. 10.1093/gerona/56.3.m146 11253156

[B21] García D. E. Alonso Ramírez J. Herrera Fernández N. Peinado Gallego C. Pérez Hernández D. G. (2019). Effect of strength exercise with elastic bands and aerobic exercise in the treatment of frailty of the elderly patient with type 2 diabetes mellitus. Endocrinol. diabetes Nutr. 66 (9), 563–570. 10.1016/j.endinu.2019.01.010 30979609

[B22] Glass D. J. (2005). Skeletal muscle hypertrophy and atrophy signaling pathways. Int. J. Biochem. and cell Biol. 37 (10), 1974–1984. 10.1016/j.biocel.2005.04.018 16087388

[B23] Gutiérrez-RegueroH. Buendía-RomeroÁ. Franco-LópezF. Martínez-CavaA. Hernández-BelmonteA. Courel-IbáñezJ. (2024). Effects of multicomponent training and HMB supplementation on disability, cognitive and physical function in institutionalized older adults aged over 70 years: a cluster-randomized controlled trial. J. Nutr. health and aging 28 (5), 100208. 10.1016/j.jnha.2024.100208 38489992

[B24] Guyatt G. H. Oxman A. D. Montori V. Vist G. Kunz R. Brozek J. (2011). GRADE guidelines: 5. Rating the quality of evidence--publication bias. J. Clin. Epidemiol. 64 (12), 1277–1282. 10.1016/j.jclinepi.2011.01.011 21802904

[B25] Halvarsson A. Franzén E. Ståhle A. (2015). Balance training with multi-task exercises improves fall-related self-efficacy, gait, balance performance and physical function in older adults with osteoporosis: a randomized controlled trial. Clin. Rehabil. 29 (4), 365–375. 10.1177/0269215514544983 25142277

[B26] Hartantri W. Andriana R. M. Satyawati R. Mikami Y. Melaniani S. (2023). Effects of a 4-week multicomponent exercise (vivifrail) on predicted maximum oxygen consumption and fatigue levels in the elderly with frailty syndrome: a randomized controlled trial. Biomol. Health Sci. J. 6, 104–110. 10.4103/bhsj.bhsj_12_23

[B27] Higgins J. P. Altman D. G. Gøtzsche P. C. Jüni P. Moher D. Oxman A. D. (2011). The cochrane Collaboration’s tool for assessing risk of bias in randomised trials. Bmj 343, d5928. 10.1136/bmj.d5928 22008217 PMC3196245

[B28] Higgins J. P. Thompson S. G. (2002). Quantifying heterogeneity in a meta‐analysis. Statistics Med. 21 (11), 1539–1558. 10.1002/sim.1186 12111919

[B29] Hoogendijk E. O. Afilalo J. Ensrud K. E. Kowal P. Onder G. Fried L. P. (2019). Frailty: implications for clinical practice and public health. Lancet London, Engl. 394 (10206), 1365–1375. 10.1016/S0140-6736(19)31786-6 31609228

[B30] Izquierdo M. (2019). Multicomponent physical exercise program: vivifrail. Nutr. Hosp. 36 (Spec No2), 50–56. 10.20960/nh.02680 31189323

[B31] IzquierdoM. Rodriguez-MañasL. SinclairA. J. (2016). Editorial: what is new in exercise regimes for frail older people - how does the erasmus vivifrail project take us forward? J. Nutr. health and aging 20 (7), 736–737. 10.1007/s12603-016-0702-5 27499307

[B32] Jackson D. White I. R. Thompson S. G. (2010). Extending DerSimonian and Laird's methodology to perform multivariate random effects meta-analyses. Statistics Med. 29 (12), 1282–1297. 10.1002/sim.3602 19408255

[B33] Labata-Lezaun N. González-Rueda V. Llurda-Almuzara L. López-de-Celis C. Rodríguez-Sanz J. Bosch J. (2023). Effectiveness of multicomponent training on physical performance in older adults: a systematic review and meta-analysis. Archives gerontology geriatrics 104, 104838. 10.1016/j.archger.2022.104838 36272227

[B34] Li Y. Li S. Weng X. Yang X. Bao J. Liao S. (2025). Effects of the Vivifrail-B multicomponent exercise program based on society ecosystems theory on physical function in community-dwelling frail older adults: a randomized controlled trial. Exp. Gerontol. 200, 112670. 10.1016/j.exger.2024.112670 39736420

[B35] Linhares D. G. Borba-Pinheiro C. J. Castro J. B. P. Santos A. Santos L. L. D. Cordeiro L. S. (2022). Effects of multicomponent exercise training on the health of older women with osteoporosis: a systematic review and meta-analysis. Int. J. Environ. Res. Public Health 19 (21), 14195. 10.3390/ijerph192114195 36361073 PMC9655411

[B36] Martínez-Velilla N. Valenzuela P. L. Sáez de Asteasu M. L. Zambom-Ferraresi F. Ramírez-Vélez R. García-Hermoso A. (2021). Effects of a tailored exercise intervention in acutely hospitalized oldest old diabetic adults: an ancillary analysis. J. Clin. Endocrinol. metabolism 106 (2), e899–e906. 10.1210/clinem/dgaa809 33150389

[B37] Mawadda I. Andriana M. Nugraheni N. Subadi I. Utomo D. N. Melaniani S. (2024). The effect of vivifrail© multicomponent exercises on handgrip strength and upper limb endurance in elderly with frailty syndrome in the nursing home: a quasi-experiment study. Bali Med. J. 13 (1), 912–916. 10.15562/bmj.v13i2.5206

[B38] Moher D. Liberati A. Tetzlaff J. Altman D. G. PRISMA Group (2009). Preferred reporting items for systematic reviews and meta-analyses: the PRISMA statement. PLoS Med. 6 (7), e1000097. 10.1371/journal.pmed.1000097 19621072 PMC2707599

[B39] Moreira N. B. Rodacki A. L. F. Costa S. N. Pitta A. Bento P. C. B. (2021). Perceptive-cognitive and physical function in prefrail older adults: exergaming *versus* traditional multicomponent training. Rejuvenation Res. 24 (1), 28–36. 10.1089/rej.2020.2302 32443963

[B40] Morley J. E. (2013). Frailty, falls, and fractures. J. Am. Med. Dir. Assoc. 14 (3), 149–151. 10.1016/j.jamda.2012.12.009 23375679

[B41] Nasreddine Z. S. Phillips N. A. Bédirian V. Charbonneau S. Whitehead V. Collin I. (2005). The Montreal cognitive assessment, MoCA: a brief screening tool for mild cognitive impairment. J. Am. Geriatrics Soc. 53 (4), 695–699. 10.1111/j.1532-5415.2005.53221.x 15817019

[B42] Nunes E. A. Colenso-Semple L. McKellar S. R. Yau T. Ali M. U. Fitzpatrick-Lewis D. (2022). Systematic review and meta-analysis of protein intake to support muscle mass and function in healthy adults. J. cachexia, sarcopenia muscle 13 (2), 795–810. 10.1002/jcsm.12922 35187864 PMC8978023

[B43] Organisation WHO (2024). Integrated care for older people (ICOPE): guidance for person-centred assessment and pathways in primary care. Available online at: https://www.who.int/publications/i/item/9789240103726.

[B44] Phillips C. Baktir M. A. Das D. Lin B. Salehi A. (2015). The link between physical activity and cognitive dysfunction in alzheimer disease. Phys. Ther. 95 (7), 1046–1060. 10.2522/ptj.20140212 25573757

[B45] Romero-GarcíaM. López-RodríguezG. Henao-MoránS. González-UnzagaM. GalvánM. (2021). Effect of a multicomponent exercise program (VIVIFRAIL) on functional capacity in elderly ambulatory: a non-randomized clinical trial in Mexican women with dynapenia. J. Nutr. health and aging 25 (2), 148–154. 10.1007/s12603-020-1548-4 33491027

[B46] Rommel C. Bodine S. C. Clarke B. A. Rossman R. Nunez L. Stitt T. N. (2001). Mediation of IGF-1-induced skeletal myotube hypertrophy by PI(3)K/Akt/mTOR and PI(3)K/Akt/GSK3 pathways. Nat. cell Biol. 3 (11), 1009–1013. 10.1038/ncb1101-1009 11715022

[B47] Sáez de Asteasu M. L. Martínez-Velilla N. Zambom-Ferraresi F. García-Alonso Y. Galbete A. Ramírez-Vélez R. (2024). Short-term multicomponent exercise impact on muscle function and structure in hospitalized older at risk of acute sarcopenia. J. cachexia, sarcopenia muscle 15 (6), 2586–2594. 10.1002/jcsm.13602 39400535 PMC11634513

[B48] Sánchez-Sánchez J. L. de Souto Barreto P. Antón-Rodrigo I. Ramón-Espinoza F. Marín-Epelde I. Sánchez-Latorre M. (2022). Effects of a 12-week vivifrail exercise program on intrinsic capacity among frail cognitively impaired community-dwelling older adults: secondary analysis of a multicentre randomised clinical trial. Age ageing 51 (12), afac303. 10.1093/ageing/afac303 36580558 PMC9799251

[B49] Soenarti S. Lestari D. I. Nugroho M. B. Lestari H. Tita Hariyanti (2024). Rehabilitation impact of vivifrail exercise program type C on the cognitive function of pre-frail elderly people in the community. Folia Medica Indones. 60 (3), 256–264. 10.20473/fmi.v60i3.62581

[B50] Song Y. Y. Sun W. J. Wang C. Tian Y. M. Liu H. Jiang Y. (2023). Effects of multicomponent exercise on quality of life, depression and anxiety among stroke survivors: a systematic review and meta-analysis. J. Clin. Nurs. 32 (21-22), 7677–7690. 10.1111/jocn.16853 37727891

[B51] Spitzer R. L. Endicott J. Robins E. (1978). Research diagnostic criteria: rationale and reliability. Archives general psychiatry 35 (6), 773–782. 10.1001/archpsyc.1978.01770300115013 655775

[B52] Stigger F. S. Zago Marcolino M. A. Portela K. M. Plentz R. D. M. (2019). Effects of exercise on inflammatory, oxidative, and neurotrophic biomarkers on cognitively impaired individuals diagnosed with dementia or mild cognitive impairment: a systematic review and meta-analysis. journals gerontology Ser. A, Biol. Sci. Med. Sci. 74 (5), 616–624. 10.1093/gerona/gly173 30084942

[B53] Walsh N. P. Gleeson M. Pyne D. B. Nieman D. C. Dhabhar F. S. Shephard R. J. (2011). Position statement. Part two: maintaining immune health. Exerc. Immunol. Rev. 17, 64–103. 21446353

[B54] Wang C. Guo X. Xu X. Liang S. Wang W. Zhu F. (2023). Association between sarcopenia and frailty in elderly patients with chronic kidney disease. J. cachexia, sarcopenia muscle 14 (4), 1855–1864. 10.1002/jcsm.13275 37300354 PMC10401549

[B55] Ward D. D. Wallace L. M. K. Rockwood K. (2021). Frailty and risk of dementia in mild cognitive impairment subtypes. Ann. neurology 89 (6), 1221–1225. 10.1002/ana.26064 33704823

[B56] Wimo A. Winblad B. Jönsson L. (2007). An estimate of the total worldwide societal costs of dementia in 2005. Alzheimer's and dementia J. Alzheimer's Assoc. 3 (2), 81–91. 10.1016/j.jalz.2007.02.001 19595921

[B57] Xue Q. L. (2011). The frailty syndrome: definition and natural history. Clin. geriatric Med. 27 (1), 1–15. 10.1016/j.cger.2010.08.009 PMC3028599 21093718

[B58] Zhang S. Zhen K. Su Q. Chen Y. Lv Y. Yu L. (2022). The effect of aerobic exercise on cognitive function in people with alzheimer's disease: a systematic review and meta-analysis of randomized controlled trials. Int. J. Environ. Res. Public Health 19 (23), 15700. 10.3390/ijerph192315700 36497772 PMC9736612

[B59] Zhu Q. Ping P. Zhang P. Ning C. Zhao Y. Yao Y. (2022). Sex hormones and physical function among the Chinese oldest-old and Centenarian women. J. Transl. Med. 20 (1), 340. 10.1186/s12967-022-03539-9 35902963 PMC9331572

