# Supplementary material for: The effects of vivifrail-based multicomponent training on physical and cognitive function in frail older adults: a systematic review and meta-analysis
Source: Front Physiol. 2025 Jul 29;16:1646833. doi: 10.3389/fphys.2025.1646833 (PMC12339439; doi:10.3389/fphys.2025.1646833)
Supplement: Supplementary file 1 [file Supplementaryfile1.docx]

Supplementary Material

**Table S1.** The details of full search strategy for systematic review and meta analysis

**Table S2.** The details of outcome data for each study

**Table S3**. The results of Kappa test

**Table S4**. Intervention protocol

**Table S5**. The results of meta-regression analyses

**Table S6.** Analysis of publication bias across studies

**Figure S1.** The summary results of the Cochrane risk of bias assessment tool

**Figure S2.** The results of the GRADE

**Figure S3.** The results of the funnel plots for all outcomes

**Table S1.** The details of full search strategy for systematic review and meta analysis

| **Database** | **Search Strategy** |
| --- | --- |
| PubMed | ("older adults" OR "older population" OR "older people" OR "Aged" OR "Elderly" OR "Aging" OR "Frailty")  AND ("Multicomponent exercise programme" OR "Multicomponent intervention" OR "Vivifrail" OR "Vivifrail exercise program" OR "Vivifrail multicomponent intervention" OR "Vivifrail programme") AND ("Short Physical Performance Battery" OR "Mini-Mental State Examination" OR "Montreal Cognitive Assessment" OR "cognitive function" OR "Barthel Index" OR "activities of daily living" OR "Handgrip" OR "functional capacity" OR "muscle function") AND "English"[Language] |
| Web of Science | (TI=("older adults" OR "older population" OR "older people" OR "Aged" OR "Elderly" OR "Aging" OR "Frailty")  OR AB=("older adults" OR "older population" OR "older people" OR "Aged" OR "Elderly" OR "Aging" OR "Frailty") AND  (TI=("Multicomponent exercise programme" OR "Multicomponent intervention" OR "Vivifrail" OR "Vivifrail exercise program" OR "Vivifrail multicomponent intervention" OR "Vivifrail programme")  OR AB=("Multicomponent exercise programme" OR "Multicomponent intervention" OR "Vivifrail" OR "Vivifrail exercise program" OR "Vivifrail multicomponent intervention" OR "Vivifrail programme")  AND  (TI=("Short Physical Performance Battery" OR "Mini-Mental State Examination" OR "Montreal Cognitive Assessment" OR "cognitive function" OR "Barthel Index" OR "activities of daily living" OR "Handgrip" OR "functional capacity" OR "muscle function")  OR AK=("Short Physical Performance Battery" OR "Mini-Mental State Examination" OR "Montreal Cognitive Assessment" OR "cognitive function" OR "Barthel Index" OR "activities of daily living" OR "Handgrip" OR "functional capacity" OR "muscle function")) |
| The Cochrane Library | #1 ("older adults"):ab,ti,kw  #2 ("older population"):ab,ti,kw  #3 ("older people"):ab,ti,kw  #4 ("Aged"):ab,ti,kw  #5 ("Elderly"):ab,ti,kw  #6 ("Aging"):ab,ti,kw  #7 ("Frailty"):ab,ti,kw  #8 #1 OR #2 OR #3 OR #4 OR #5 OR #6 OR #7  #9 ("Multicomponent exercise programme"):ab,ti,kw  #10 ("Multicomponent intervention"):ab,ti,kw  #11 ("Vivifrail"):ab,ti,kw  #12 ("Vivifrail exercise program"):ab,ti,kw  #13 ("Vivifrail multicomponent intervention"):ab,ti,kw  #14 ("Vivifrail programme"):ab,ti,kw  #15 #9 OR #10 OR #11 OR #12 OR #13 OR #14  #16 ("Short Physical Performance Battery"):ab,ti,kw  #17 ("Mini-Mental State Examination"):ab,ti,kw  #18 ("Montreal Cognitive Assessment"):ab,ti,kw  #19 ("cognitive function"):ab,ti,kw  #20 ("Barthel Index"):ab,ti,kw  #21 ("activities of daily living"):ab,ti,kw  #22 ("Handgrip"):ab,ti,kw  #23 ("functional capacity"):ab,ti,kw  #24 ("muscle function"):ab,ti,kw  #25 #16 OR #17 OR #18 OR #19 OR #20 OR #21 OR #22 OR #23 OR #24  #26 "English":la  #8 AND #15 AND #25 AND #26 |
| EBSCOhost | S1 TI ("multicomponent exercise" OR "Vivifrail" OR "exercise programme" OR "exercise intervention" OR "physical activity" OR "physical training")  S2 TI ("older adults" OR "older population" OR "Aged" OR "Elderly" OR "Aging" OR "Frailty")  S3 TS=("Short Physical Performance Battery" OR "Mini-Mental State Examination" OR "Montreal Cognitive Assessment" OR "cognitive function" OR "Barthel Index" OR "activities of daily living" OR "Handgrip" OR "functional capacity" OR "muscle function")  S4 Narrow by Language: - English  S5 S1 AND S2 AND S3 AND S4 |
| Embase | 1 'multicomponent exercise' OR 'Vivifrail' OR 'exercise programme' OR 'physical activity' OR 'physical training' OR 'aerobic exercise' OR 'resistance training' OR 'interval training' OR 'high-intensity interval training'  2 'Short Physical Performance Battery' OR 'Mini-Mental State Examination' OR 'Montreal Cognitive Assessment' OR 'cognitive function' OR 'Barthel Index' OR 'activities of daily living' OR 'Handgrip' OR 'functional capacity' OR 'muscle function'  3 'English'  4 1 AND 2 AND 3 |

**Table S2.** The details of outcome data for each study

| Study | Outcome | Group | N | Mean_change_ | SD_change_ |
| --- | --- | --- | --- | --- | --- |
| Casas-Herrero et al. (2022) | SPPB | EX | 88 | 1.07 | 2.076649563 |
|  |  | UC | 100 | -0.33 | 1.864715447 |
|  | MOCA | EX | 88 | 2.05 | 5.85237604 |
|  |  | UC | 100 | -0.13 | 4.787782905 |
|  | ADL | EX | 88 | 0.99 | 11.30358114 |
|  |  | UC | 100 | -0.1 | 10.33153153 |
|  | Handgrip | EX | 88 | 0.35 | 3.634136735 |
|  |  | UC | 100 | -0.7 | 3.52784003503416 |
|  |  |  |  |  |  |
| Sánchez-Sánchez et al. (2022) | SPPB | EX | 88 | 0.54 | 3.138572634 |
|  |  | UC | 100 | -0.46 | 5.039771479 |
|  | MOCA | EX | 88 | 2.04 | 9.203333289 |
|  |  | UC | 100 | -0.31 | 5.545598264 |
|  | Handgrip | EX | 88 | 2.86 | 8.141410217 |
|  |  | UC | 100 | -0.73 | 2.666606825 |
|  |  |  |  |  |  |
| Chen et al. (2023) | SPPB | EX | 44 | 0.5 | 1.25 |
|  |  | UC | 60 | -2 | 0.943729304 |
|  | MMSE | EX | 44 | 3 | 1.952562419 |
|  |  | UC | 60 | 2 | 1.5612495 |
|  | Handgrip | EX | 44 | -0.4 | 6.455230437 |
|  |  | UC | 60 | -0.1 | 7.05336799 |
|  | ADL | EX | 44 | 10 | 2.5 |
|  |  | UC | 60 | 0 | 3.75 |
|  |  |  |  |  |  |
| Romero-García et al. (2021) | SPPB | EX | 33 | 0.25 | 0.746793144 |
|  |  | UC | 28 | 0.03 | 0.740810367 |
|  | Handgrip | EX | 33 | 1.67 | 4.105252733 |
|  |  | UC | 28 | -1.87 | 3.320180718 |
|  |  |  |  |  |  |
| Gutiérrez-Reguero et al. (2024) | SPPB | EX | 17 | 2.97 | 1.731003743 |
|  |  | UC | 19 | 0.25 | 1.75316815 |
|  | MMSE | EX | 17 | 2.91 | 2.402010811 |
|  |  | UC | 19 | -1.13 | 2.440909772 |
|  | Handgrip | EX | 17 | 1.15 | 3.199439502 |
|  |  | UC | 19 | -1.12 | 2.666060442 |
|  | ADL | EX | 17 | 8.13 | 11.79610977 |
|  |  | UC | 19 | -0.62 | 12.23068195 |
|  |  |  |  |  |  |
| Dobarro et al. (2021) | SPPB | EX | 29 | 1.21 | 2.1 |
|  |  | UC | 27 | 0.97 | 1.9 |
|  |  |  |  |  |  |
| Li et al. (2025) | SPPB | EX | 30 | 1.03 | 0.906697303 |
|  |  | UC | 29 | 0.14 | 0.720208303 |
|  | Handgrip | EX | 30 | 0.71 | 2.190068492 |
|  |  | UC | 29 | -0.27 | 2.120117921 |
|  | ADL | EX | 30 | -1.3 | 2.665126639 |
|  |  | UC | 29 | 0.28 | 4.331039136 |
|  |  |  |  |  |  |
| Sáez de Asteasu et al. (2024) | SPPB | EX | 64 | 1.3 | 2.021677444 |
|  |  | UC | 66 | 0.32 | 4.027162064 |
|  | Handgrip | EX | 64 | 0.94 | 3.322756987 |
|  |  | UC | 66 | −0.24 | 3.416985993 |
|  |  |  |  |  |  |
| Martínez-Velilla et al. (2021) | SPPB | EX | 54 | 2.6 | 2.198225272 |
|  |  | UC | 49 | -0.1 | 2.088892567 |
|  | MMSE | EX | 54 | 1.7 | 2.381410712 |
|  |  | UC | 49 | 0.1 | 2.262966948 |
|  | Handgrip | EX | 54 | 1.8 | 2.747781591 |
|  |  | UC | 49 | -0.7 | 2.611115709 |
|  | ADL | EX | 54 | 3.7 | 10.96668598 |
|  |  | UC | 49 | -4.8 | 11.6629835 |
|  |  |  |  |  |  |
| Mawadda et al. (2021) | SPPB | EX | 25 | 1.24 | 1.478242199 |
|  |  | UC | 25 | 0.36 | 2.331608887 |
|  | Handgrip | EX | 25 | 3.46 | 3.67 |
|  |  | UC | 25 | -0.29 | 1.66 |
|  |  |  |  |  |  |
| Soenarti et al. (2024) | MMSE | EX | 17 | 3.81 | 4.32 |
|  |  | UC | 10 | 1.1 | 1.45 |
|  |  |  |  |  |  |
| Courel-Ibáñez et al. (2020) | SPPB | EX | 12 | 2.2 | 1.731275542 |
|  |  | UC | 12 | -0.91 | 0.0865637771154458 |
|  | Handgrip | EX | 12 | 2.4 | 2.345091416 |
|  |  | UC | 12 | -0.91 | 1.825708754 |

**Table S3**. The results of Kappa test

|  | Value | 95%CI |
| --- | --- | --- |
| Measure of agreement (Kappa) | 0.84 | 0.59 to 1.00 |
| N of vaild cases | 25 | / |

**Table S4**. Intervention protocol

| Study | Aerobic |  | Progressive resistance |  | Balance |  | Flexibility |  | Duration |
| --- | --- | --- | --- | --- | --- | --- | --- | --- | --- |
|  | Content | Frequency | Content | Frequency | Content | Frequency | Content | Frequency |  |
| Casas-Herrero et al. (2022) | Walking. | 5 d/wk | Upper-body: Initial load was set at 0.5 kg (dumbbells), gradually increased in 0.5 kg increments. Lower-body: Free weight repetitions and gradually increased in 0.5-kg increments using ankle weights. | 3 d/wk | NR | 3 d/wk | NR | 3 d/wk | 12 weeks |
| Chen et al. (2023) | Walking | 5 d/wk | Starting at 30% of one repetition maximum, with 10–12 per groups and 3–5 groups. | 7 d/wk | Standing on double, front, and back feet and other Chinese traditional activities. | 3 d/wk | NR | NR | 12 weeks |
| Courel-Ibáñez et al. (2020) | NR | NR | NR | NR | NR | NR | NR | NR | 4 weeks |
| Dobarro et al. (2021) | NR | NR | NR | NR | NR | NR | NR | NR | 6 months |
| Gutiérrez-Reguero et al. (2024) | Walking. | 2 d/wk | Handgrip, biceps curl, squat  and knee extension. | 3 - 5 d/wk | Walking on toes and heels,  around small obstacles, and stepping | 3 - 5 d/wk | Arm and hamstring  stretching | 3 - 5 d/wk | 12 weeks |
| Li et al. (2025) | Walking. | 5 d/wk | Lift a bottle;Squeeze a ball; Pretend to sit. | 5 d/wk | Walking on tiptoes and heels Stand up | 5 d/wk | Arm stretching in a chair hold this position for 10 s | 5 d/wk | 12 weeks |
| Martínez-Velilla et al. (2021) | Walking. | 5 - 7d/wk | Bench press, squads, leg press and knee extension | 5 - 7d/wk | Semi-tandem foot standing, line walking, stepping practice, walking with small obstacles, proprioceptive exercises on unstable surfaces, altering the base of support, and weight transfer from one leg to the other. | 5 - 7d/wk | NR | NR | 12 weeks |
| Mawadda et al. (2021) | Walking. | 5 d/wk | 2 sets of 10 repetitions with a load of 30× maximum repetitions. | 3 d/wk | Holding 10 counts for each movement | 3 d/wk | Flexibility for each training session, 2 sets of 3 repetitions, holding 10 seconds for each position. | 3 d/wk | 12 weeks |
| Romero-García et al. (2021) | Walking. | 5 d/wk | Started with 2 sets of 10 repetitions and progressed until 3 sets of 12-15 repetitions with 1-3 min break between exercises. | 3 - 4 d/wk | Multitask walks (throwing a balloon) and obstacles (five) walks for 8 times, progressed with position changes or closed eyes. | 3 d/wk | Remained position 2 sets of 3 repetitions for 10-12 seconds. | 5 d/wk | 12 weeks |
| Sáez de Asteasu et al. (2024) | NR | NR | Bench press, squads, leg press and knee extension | 3 - 5 d/wk | NR | NR | NR | NR | 3 - 5 days |
| Sánchez-Sánchez et al. (2022) | Walking. | 5 d/wk | Sitting arm curl, ball squeeze or towel roll-up (handgrip), shoulder extension with elastic band, sitting calf-raise, sitting knee extension, sitting hip abduction. | 3 d/wk | Tandem walk, one-leg standing toes to heel roll, tiptoe walking, heel walking, walking overcoming obstacles, multi-direction walking, walking with exterNRl distractors. | 3 d/wk | Arms stretching, back shoulder stretching,  cervical region stretching, dorsal-plantar flexion,  sitting back muscular chain stretchin | 3 d/wk | 12 weeks |
| SoeNRrti et al. (2024) | Walking. | 2 d/wk | Sitting arm curl, ball squeeze or towel roll-up (handgrip), shoulder extension with elastic band, standing calf-raise, sitting knee extension, hip abduction, knee flexion, chair sit to stand. | 2 d/wk | Tandem walk, one-leg standing, toes to heel roll, tiptoe walking, heel walking, walking overcoming obstacles, multi-direction walking | 2 d/wk | Arms stretching, back shoulder stretching, cervical region stretching, dorsal-plantar flexion, sitting back muscular chain stretching | 2 d/wk | 12 weeks |

**Table S5.**The results of meta-regression

|  | β (95% CI) |  |  |
| --- | --- | --- | --- |
| Outcomes | Design | Age | Duration |
| SPPB | 0.25 (-0.55 to 1.06) | 0.27 (-0.39 to 0.93) | 0.25 (-0.79 to 1.29) |
| Cognition | 0.20 (-0.72 to 1.12) | 0.22 (-0.63 to 1.08) | / |
| ADL | 0.24 (-0.71 to 1.19) | / | / |
| Hand grip | 0.24 (-0.58 to 1.08) | 0.25 (-0.45 to 0.95) | 0.24 (-0.43 to 0.91) |

**Table S6.** Analysis of publication bias across studies

| Outcome | Egger's test | | | | Duval and Tweedie’s trim and fill | | | | |
| --- | --- | --- | --- | --- | --- | --- | --- | --- | --- |
|  | β | SE | 95% CI | p value | Studies trimmed | Observed effect size | | Adjusted effect size | |
|  |  |  |  |  |  | Hedges’g | 95% CI | Hedges’g | 95% CI |
| SPPB | -0.19 | 0.52 | -1.38, 1.0 | 0.72 | 0 | 0.30 | 0.03, 0.57 | 0.34 | 0.08, 0.60 |
| Cognitive function | -0.10 | 0.21 | -0.70, 0.49 | 0.64 | 0 | 0.34 | 0.19, 0.49 | 0.34 | 0.18, 0.50 |
| ADL | -0.89 | 1.54 | -5.80, 4.01 | 0.60 | 0 | 0.27 | 0.10, 0.44 | 0.27 | 0.10, 0.43 |
| Hand grip | -0.11 | 0.31 | -0.84, 0.60 | 0.71 | 0 | 0.45 | 0.1, 0.8 | 0.45 | -0.01, 0.92 |


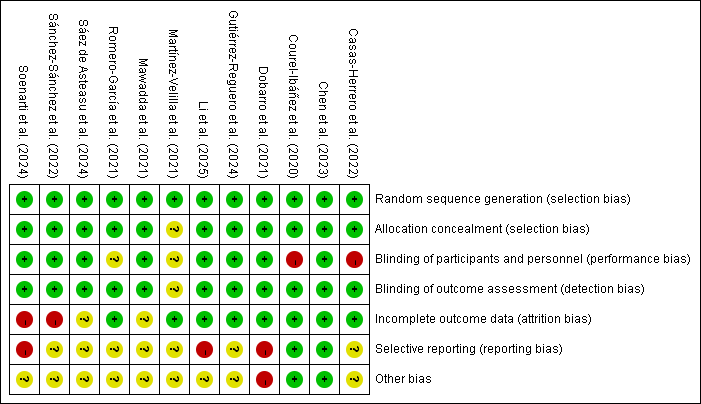


**Figure S1.** The summary results of the Cochrane risk of bias assessment tool


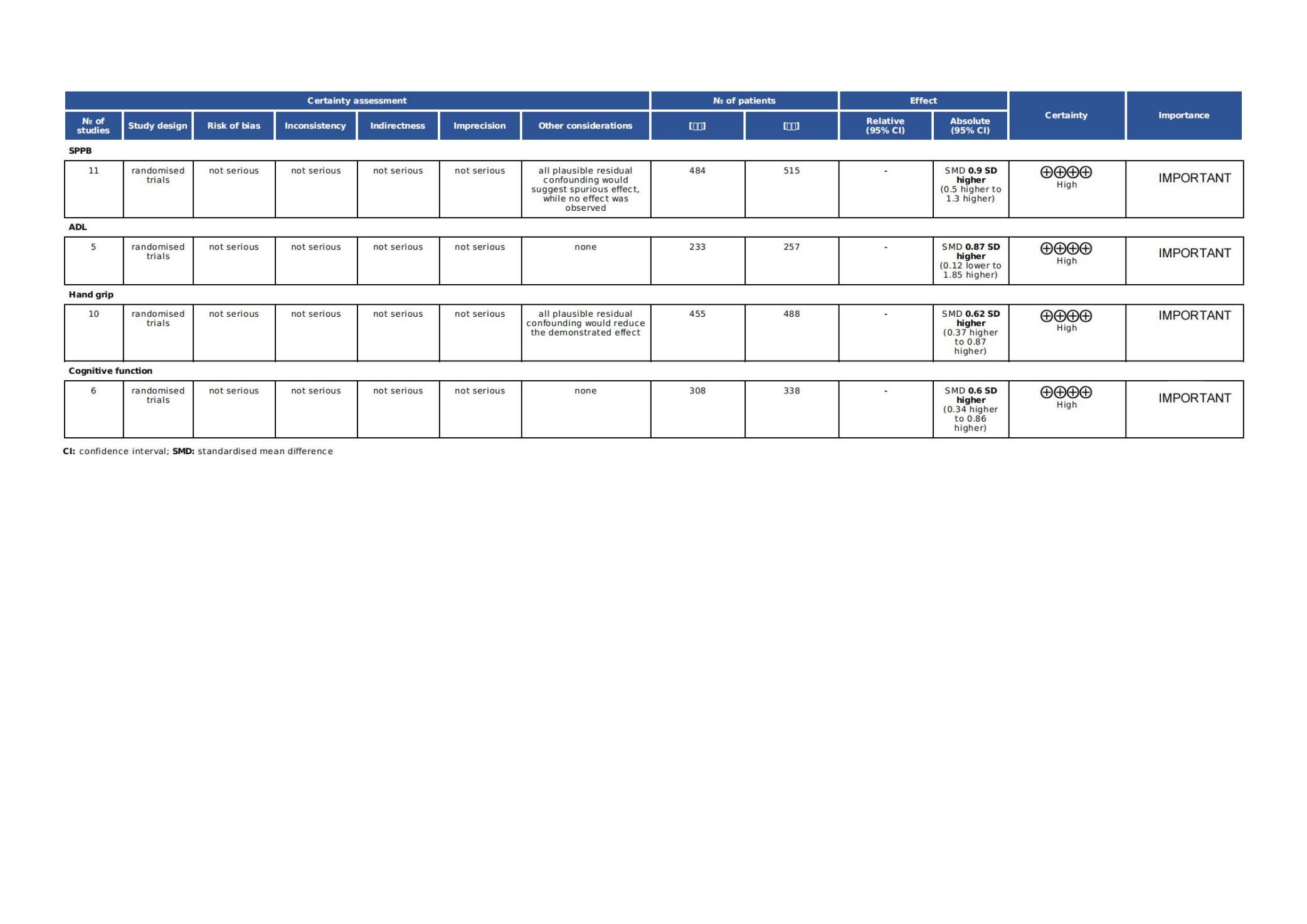


**Figure S2.** The results of the GRADE


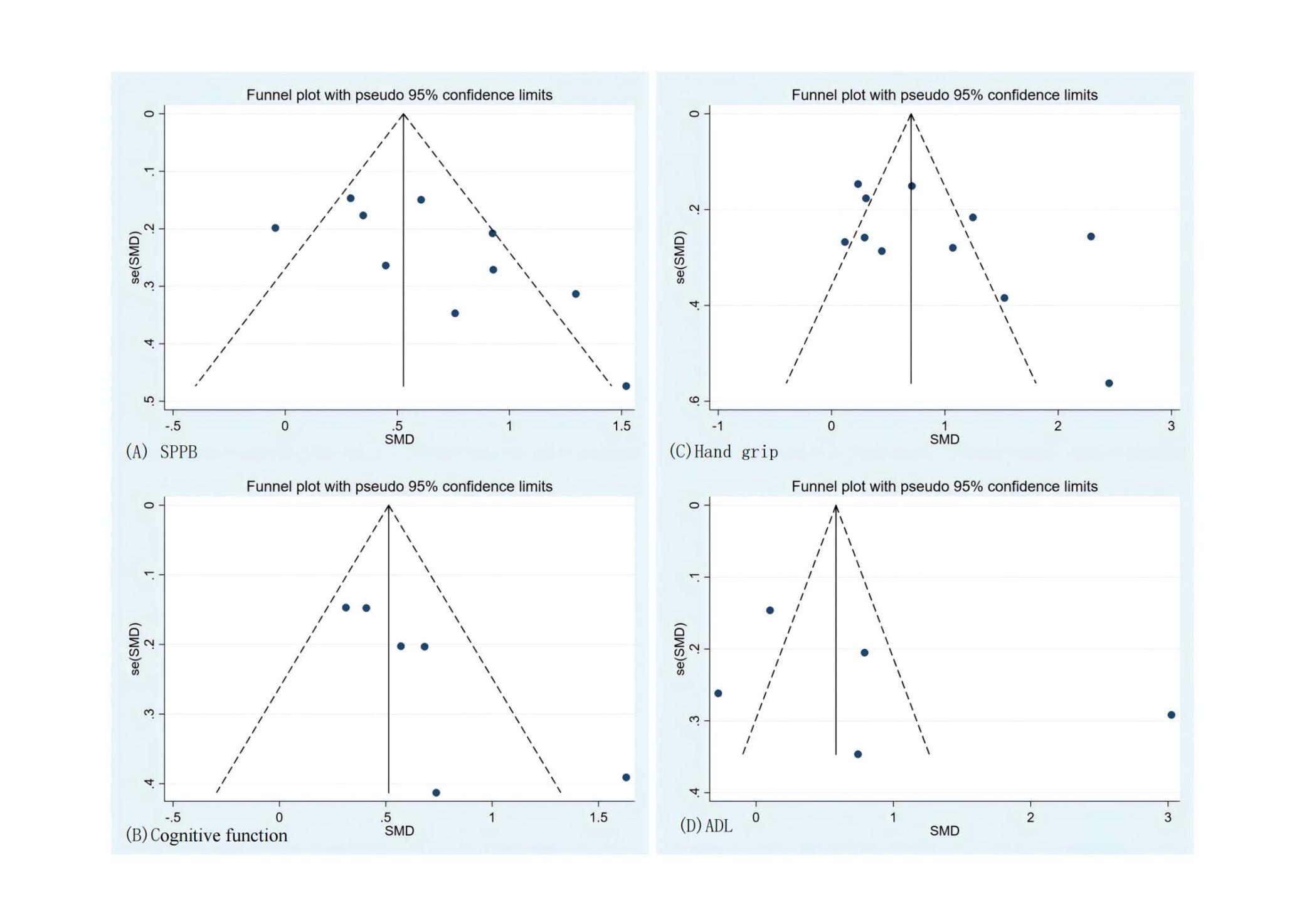


**Figure S3.** The results of the funnel plots for all outcomes
